# Intestinal obstruction caused by a laxative drug (Psyllium): A case report and review of the literature^☆^

**DOI:** 10.1016/j.ijscr.2018.10.001

**Published:** 2018-10-08

**Authors:** Ashraf F. Hefny, Adel Z. Ayad, Nikolay Matev, Masoud O. Bashir

**Affiliations:** a Department of Surgery, College of Medicine and Health Sciences, UAE University, Al-Ain, United Arab Emirates; b Department of Emergency, Al Rahba Hospital, Abu Dhabi, United Arab Emirates; c Department of Radiology, Al Rahba Hospital, Abu Dhabi, United Arab Emirates; d Department of Surgery, Al Rahba Hospital, Abu Dhabi, United Arab Emirates

**Keywords:** Case report, Constipation, Gastrointestinal tract, Laxatives, Psyllium

## Abstract

**Introduction:**

Psyllium in granular dosage form is used as an over – the counter safe laxative drug. It has a considerable hygroscopic propriety which allows it to retain water and expands rapidly to become many times of its original size.

**Presentation of the case:**

21-year-old man presented to the Emergency Department complaining of lower abdominal pain and constipation for five days. He was discharged home on Psyllium for treatment of the constipation. Two days later, he returned to the Emergency Department complaining of increased abdominal distension without passing any stools. The patient was diagnosed to have an incomplete intestinal obstruction resulting from ingesting Psyllium husks without adequate amount of fluids. The patient was treated conservatively.

**Discussion:**

Psyllium has a reputation of being safe and effective medication for patients suffering from chronic constipation, especially in elderly. The patient who ingested Psyllium husks as a herbal medication did not receive adequate amount of fluids. Consequently, he developed intestinal obstruction.

Similar cases have been reported in the English literature including cases of esophageal obstruction in elderly patients and following gastric banding operations. Furthermore, Psyllium administration with oral contrast in computed tomography enterography may precipitate bowel obstruction in the presence of organic obstruction or post-operative ileus.

**Conclusion:**

Psyllium can worsen the constipation if not taken appropriately. It is important to instruct patients who are receiving psyllium ingredients to drink a good amount of fluids to avoid the development of bowel obstruction especially in long-term use of such laxatives.

## Highlights

• Elderly patients receiving psyllium for treatment of constipation must drink a good amount of fluid.
• It is advisable to avoid bulk laxative treatment in presence of organic bowel obstruction or ileus.
• Oral contrast materials that contain Psyllium can cause bowel obstruction in some patients.

## 1 Introduction

Psyllium seed husks (also known as Ispaghula or psyllium) are natural derivatives from the Plantago ovata plant [1,2]. Psyllium husks are indigestible and 65% of their constituents are insoluble polysaccharides with a powerful ability to form a gel in water [2]. They have also a considerable hygroscopic propriety which allows for the original material to retain water and expand rapidly to many times its original size [3]. Because of these properties, Psyllium husks have become an increasingly popular food supplement used to help in weight reduction (by producing feeling of fullness and satiety) and promote natural bowel movement (by increasing the bulk of the stool) [4].

Psyllium in granular dosage form is used as an over – the counter safe laxative drug. It is known for having a marked bulking effect on the stools and it should be swallowed with adequate amount of fluids [5,6]. Psyllium is used also in the treatment of fecal incontinence (because of its ability to retain water), hemorrhoids, ulcerative colitis, and hyperlipidemia [2].

Psyllium granular form is generally safe, yet, it has been reported that administration of the drug without adequate fluids intake can result in esophageal obstruction especially in elderly people [[7], [8], [9]]. Mechanical obstruction can also occur in predisposed groups of patients as those with congenital intestinal anomalies, paralytic ileus, and in patients after gastric bandage operations [5].

Herein, we report a case of incomplete intestinal obstruction in a young man due to ingestion of Psyllium husks and we reviewed the English literature on this important topic. The Ethical Committee at our institution has approved this research project. The approval reference is ARH/REC091.

The presented article has been reported in line with the SCARE criteria [10].

## 2 Case report

**Fig. 1:**
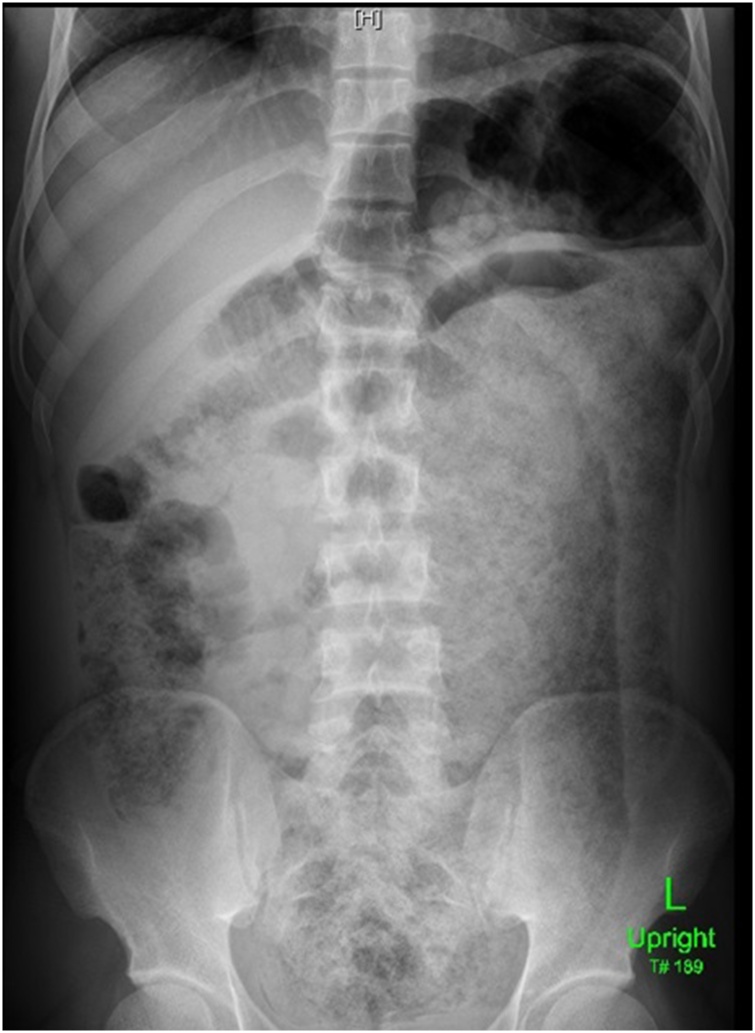
Plain erect abdominal X-ray showing huge fecal loading of the whole colon.

21-year-old man presented to the Emergency Department complaining of a lower abdominal pain and constipation for 5 days. He had neither a history of chronic medical illness nor abdominal operations. His vital signs were normal, and the abdomen was mildly distended. X-ray of the abdomen was normal. The patient was diagnosed to have a constipation and was discharged home on Psyllium (ispaghula husk) sachets 7 g twice daily as a laxative. Two days later, the condition of the patient became worse and he returned to the Emergency Department complaining of increased abdominal distension without passing any stools, yet, he was passing flatus. On examination, the vital signs were normal. His abdomen was markedly distended but soft and lax. The intestinal sounds were audible. P/R examination was normal with soft fecal matter. Repeated abdominal X-ray showed a huge fecal loading filling the whole colon with no gas fluid levels (Fig. 1).

**Fig. 2:**
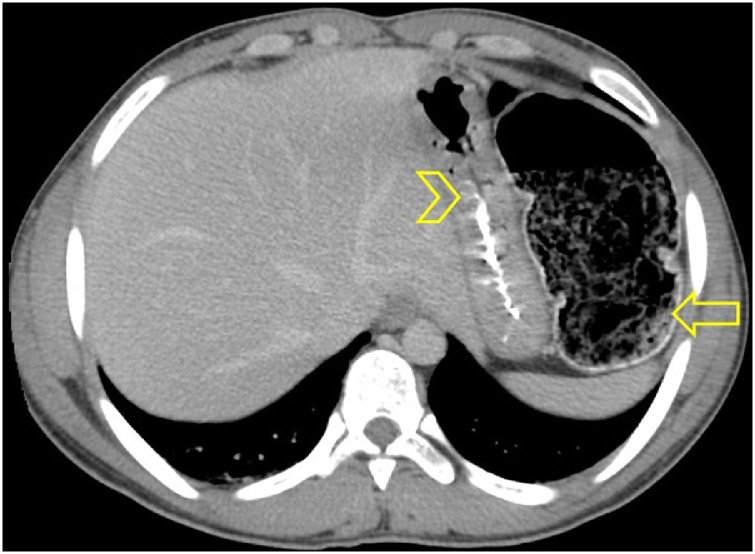
Axial CT scan of the abdomen showing distended colon (arrow) and compressed stomach (arrow head) by extrinsic compression of the colon.

**Fig. 3:**
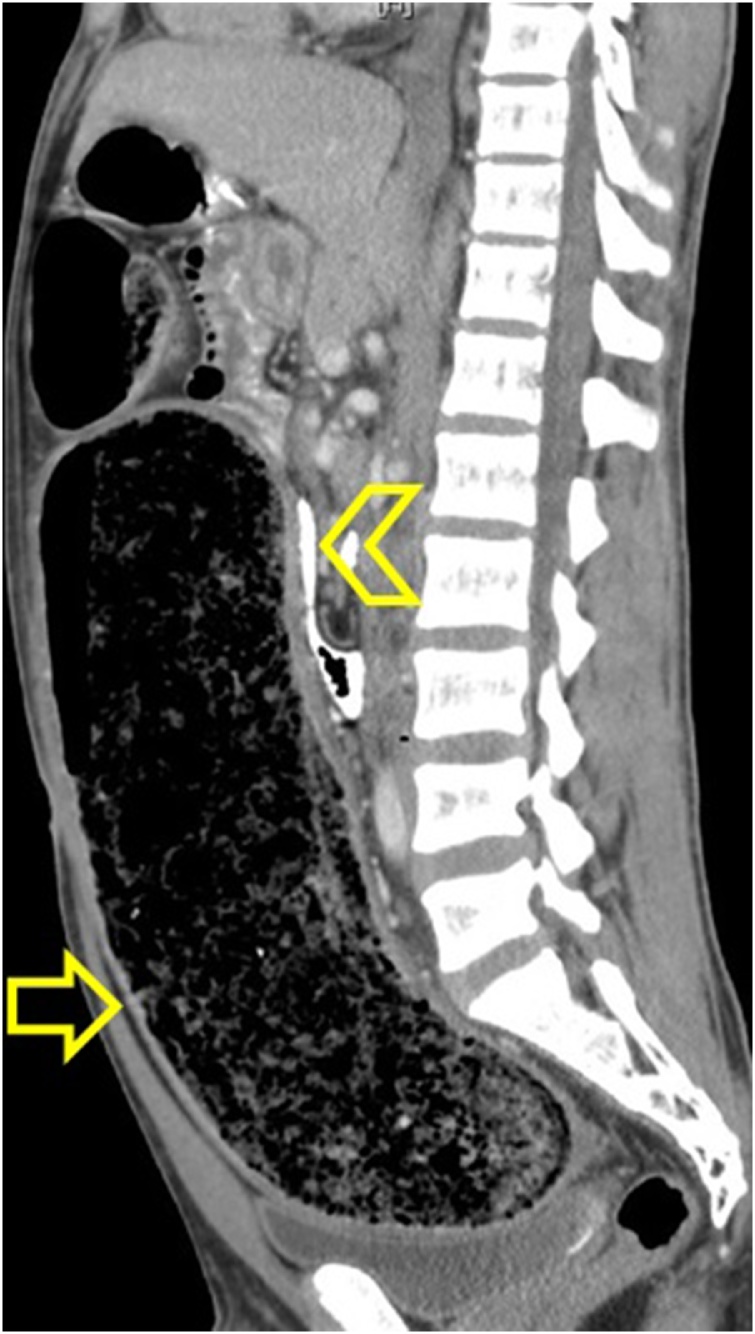
CT scan sagittal view showing a distended sigmoid colon (arrow) and a compressed bowel loops (arrow head).

Computed tomography (CT) scan of the abdomen with oral contrast has shown marked dilatation of the whole colon (Fig. 2) especially the sigmoid colon which was markedly distended and filled with gas and fecal matter (Fig. 3). The patient was diagnosed to have an incomplete intestinal obstruction and was admitted to the surgical department for further management. During his stay in the hospital, the patient admitted that prior to the onset of the constipation he had ingested psyllium husks as herbal medicine for the purpose of weight control and health promotion. He ingested the husks without adequate amount of fluids because he was fasting in Ramadan (during fasting hours, no eating or drinking fluids are allowed). While in the hospital, the patient received repeated enemas. He passed a huge amount of fecal matter and the intestinal obstruction was relieved. The patient was discharged home two days later without any operative intervention.

## 3 Discussion

Psyllium has a reputation of being a safe and effective form of medication. It is widely used in patients suffering from chronic constipation especially in elderly. The drug should be administered with large amount of fluids. The Food and Drug Administration (FDA) considered the use of Psyllium as a laxative in granular form is not generally recognized as safe due to reports of esophageal obstruction related to its usage [7].

Our patient who ingested Psyllium husks as a herbal medication did not drink the required amount of fluids because he was fasting. When he attended the Emergency Department for the first time, again, he received Psyllium sachets as a treatment for the constipation. Consequently, the patient developed incomplete bowel obstruction. Fecal impaction was a far possibility as the patient was a young adult, with no history of chronic constipation, and P/R examination has shown absence of hard fecal matter. Repeated enemas helped in evacuation of the stool and relieve the obstruction.

Intestinal obstruction can result from Psyllium usage in patients who are habitually drinking very little water or other fluids.

Fisher [8] reported a case of esophageal obstruction in 74-year- old lady following psyllium seed ingestion. He stated that “Psyllium seeds and similar bulk producers are dangerous substances, especially in elder people”. Since then, many reports have supported this opinion. Geriatric patients with chronic constipation are receiving Psyllium and other colloid laxatives for treatment, yet, they may not drink adequate amount of fluids during the treatment which can result in esophageal obstruction [[11], [12], [13], [14], [15]].

Florian et al. [5] reported a case of bolus obstruction of the esophagus due to Psyllium usage after gastric banding operations. Restriction of liquid intake around the meals was advised in gastric banding operations to avoid a rapid passage of food through the Lap-Band which resulted in esophageal obstruction in the patient [5].

Psyllium and other hydrophilic colloid laxatives administration may be very risky in the presence of organic obstruction or in the post-operative ileus. The drug increases gut distension due to progressive swelling of colloid bolus. When distension reaches a certain critical level, the bowel is unable to pass the bolus which may precipitate intestinal obstruction and spontaneous rupture of the bowel [1,16].

Psyllium preparations can form Pharmacobezoar (tightly packed mass) that most often occur, as do bezoars of any other type, whenever there is altered motility or abnormal anatomy of the gastrointestinal tract [3,17,18]. Agha et al. [19] have reported a case of massive bezoar in the right colon due to Psyllium seed husks causing complete gastric outlet obstruction by extrinsic compression.

Recently, Psyllium has been used in the oral contrast of computed tomography enterography (CTE) and magnetic resonance enterography (MRE) to allow adequate distension of the bowel which increases the diagnostic accuracy of the CTE and MRE. This was mainly due to the fact that, Psyllium is inexpensive, easy to prepare, and well-tolerated by the patients. Chen et al. [20] presented four cases who developed a small bowel obstruction following the usage of Psyllium as an oral contrast in CTE and MRE. Only one of them needed operative intervention while the other three were treated conservatively. The authors concluded that, the presence of small bowel strictures is a risk factor for obstruction following the usage of Psyllium in the oral contrast [20].

Most of the cases with bowel obstruction caused by pharmacobezoar were diagnosed and treated by gastrointestinal tract endoscopy [5]. Conservative therapy may relieve the obstruction with colloid bolus, though, some cases needed surgical intervention to relieve the obstruction and manage complications such as bowel perforation [1].

## 4 Conclusion

Psyllium can worsen the constipation if not taken appropriately. It is important to instruct patients who are receiving psyllium ingredients to drink a good amount of fluids to avoid the development of bowel obstruction especially in long-term use of such laxatives. It is advisable to avoid colloid laxative treatment in presence of paralytic ileus. Radiologists should be aware of the potential complications of using oral contrast material containing Psyllium especially when organic bowel obstruction is suspected.

## Conflict of interest

There is no conflict of interest among all the authors.

## Sources of funding

Self-funded.

## Ethical approval

The Ethical Committee at our institution has approved this research project. The approval reference is ARH/REC091.

## Consent

Written informed consent was obtained from the patient for publication of this case report and accompanying images. A copy of the written consent is available for review by the Editor-in-Chief of this journal on request.

## Author contribution

Hefny A: study concept, data collection, interpretation, writing the first draft, editing the paper, and approved the final version.

Adel Z Ayad: study concept, interpretation, editing the paper, and approved the final version.

Nikolay Matev: study concept, interpretation, editing the paper, and approved the final version.

Masoud O Bashir: study concept, interpretation, editing the paper, and approved the final version.

## Guarantor

All the authors are responsible for the article.

## Provenance and peer review

Not commissioned, externally peer reviewed.
